# Preoperative Care Clinic Improves Survival for Patients Undergoing Free‐Flap Reconstruction

**DOI:** 10.1002/ohn.1373

**Published:** 2025-08-04

**Authors:** Randall J. Harley, Stephen A. Esper, Tracy Z. Cheng, Soukaina Eljamri, Preetha Velu, Mario G. Solari, Shaum S. Sridharan, Mark Kubik, Seungwon Kim

**Affiliations:** ^1^ Department of Otorhinolaryngology–Head and Neck Surgery University of Pennsylvania Philadelphia Pennsylvania USA; ^2^ Department of Anesthesia, Center of Perioperative Care UPMC Pittsburgh Pennsylvania USA; ^3^ Department of Otolaryngology and Head and Neck Surgery UPMC Pittsburgh Pennsylvania USA; ^4^ School of Medicine University of Pittsburgh Pittsburgh Pennsylvania USA; ^5^ Department of Plastic Surgery UPMC Pittsburgh Pennsylvania USA

**Keywords:** free‐flap reconstruction, head and neck cancer, hospital‐free days, length of hospital stay, preoperative care, quality improvement, squamous cell carcinoma, survival

## Abstract

**Objective:**

This study aims to evaluate whether perioperative care improves postoperative outcomes for head and neck reconstruction patients with preexisting health conditions.

**Study Design:**

Retrospective cohort study.

**Setting:**

Single tertiary academic center between 2013 and 2021.

**Methods:**

This study included adult patients who underwent free‐flap reconstruction for head and neck cancer. Patients who received perioperative care were compared with patients who received our institution's standard of care. Comorbid health conditions were measured using the Charlson comorbidity index (CCI) excluding solid tumors. Primary outcomes were major and minor complications, length of hospital stay (LOS), days in intensive care unit (ICU), discharge to acute/subacute facility, hospital‐free days, and overall survival (OS). Interaction models were specified to evaluate the impact of preoperative care with respect to CCI.

**Results:**

Of the 148 patients included, 83 received perioperative care and 65 received institutional standard of care (mean [SD]: age, 62.1 [10.8]; male, 100 [67.6%]; CCI ≥ 4, 43 [29.1%]). Patients with higher CCI who received perioperative care spent fewer days in the hospital (CCI 3: coefficient [β], −5.50; *P* = .012 and CCI ≥ 4: β, −6.41; *P* = .022) and ICU (CCI 3: β, −2.90; *P* = .002 and CCI ≥ 4: β, −6.54; *P* = .001), gained more hospital‐free days (CCI ≥ 4: β, 17.00; *P* = .002), and had improved OS (CCI ≥ 4: adjusted hazard ratio [aHR], 0.14; *P* = .023). Perioperative care was not significantly associated with lower rates of major and minor complications or placement at a facility.

**Conclusion:**

Perioperative care provides a robust benefit for patients with medical comorbidities undergoing head and neck reconstruction, but this effect incrementally decreases for healthier patients.

Microvascular free‐flap reconstruction is the current standard of care for the majority of head and neck cancers that involve large defects with functional implications. Unfortunately, these lengthy procedures are associated with relatively high rates of adverse events and extended hospital stays.^1^ Major and minor complication rates have been cited to be around 30% and 6%, respectively, whereas rates of death before leaving the hospital range from 0.7% to 3%.^2^, ^3^, ^4^, ^5^ In recent studies, the median length of hospital stay (LOS) has ranged from 7 to 10 days and can be markedly increased in the setting of postoperative complications and placement/insurance issues.^4^, ^6^, ^7^ Comorbidities such as cardiovascular disease, kidney disease, diabetes, tobacco use, alcohol abuse, poor nutritional intake, and history of radiation therapy have been shown to negatively impact all of these outcomes.^5^, ^8^

Previous studies have described optimization before head and neck free‐flap reconstructive surgery.^1^, ^9^, ^10^ Guidelines from the Enhanced Recovery After Surgery (ERAS) Society suggest techniques such as carbohydrate treatment and pharmacologic thromboprophylaxis as part of standard preoperative care.^1^ Although standardized guidelines currently exist, the implementation of these guidelines is often variable and institution‐specific. Additionally, although several tertiary cancer centers incorporate a multidisciplinary approach to free‐flap reconstruction, there is only a single study from Switzerland showing that multidisciplinary preoperative evaluation of head and neck patients shortens hospital length of stay by 4 days, decreases complication severity, and reduces cost per case.^11^ A study of this kind in the United States for head and neck free‐flap patients has not previously been reported in literature.

The University of Pittsburgh Medical Center offers a multidisciplinary “prehabilitation” clinic called the Center for Perioperative Care (CPC). The goal of the CPC is to optimize patients' health before surgery to improve patient‐centered postoperative outcomes such as LOS and survival. Recommendations are made regarding preoperative and postoperative management of patients' medical comorbidities, nutritional optimization, and pharmacologic thromboprophylaxis. The present study focuses on patients undergoing free‐flap reconstruction for head and neck cancer. We hypothesize that patients with the highest burden of medical comorbidities will receive the greatest benefit from prehabilitation.

## Methods

### Institutional Standard of Care and CPC

The UPMC CPC, which exists as a series of outpatient clinics and an inpatient service, focuses not only on cardiac and pulmonary assessment but has a holistic view of the patient. The primary aim of the CPC is to reduce perioperative risk by helping to modify patients' lifestyles and reengaging them in their community care.^12^ Utilizing the published machine‐learning predictive algorithm and protocol‐driven care based on a risk model tested and validated on ~1.5M patients at UPMC,^13^ the program's main goals are to (1) identify patients who are at high‐risk for short‐ and long‐term poor surgical outcomes, encouraging a “surgical pause” for the opportunity to mitigate risk; (2) design a program that provides a comprehensive menu of services aimed at improving both physiologic and psychosocial conditions that contribute to vulnerability of high risk, before their nonurgent or even urgent surgical procedure; (3) provide at‐risk patients with a supportive “surgery coach,” specifically trained in surgical preparation, who mentors patients, readying them for their surgical experience; (4) engage an anesthesiologist‐directed multidisciplinary team comprised of surgeons, primary care providers, and physician specialists all working in concert with the patient in a shared decision‐making approach to determine a patient's options and candidacy for surgery, which includes an anesthesiology led detailed goals of care discussions with patients during which best, worst, and most likely case scenarios for operative or nonoperative approaches, during which the patients' values are shared.

A standard preoperative visit includes a history and physical, labs, assessment to determine if cardiopulmonary status is functional, and day of surgery evaluation by anesthesiology. More complex evaluations of health comorbidities are generally coordinated through primary care. A CPC visit includes the aforementioned items as well as expanded blood work, including diabetes and nutrition labs, expanded cardiopulmonary evaluation including options for in‐clinic chest x‐ray, electrocardiogram, or transthoracic echocardiogram,^14^, ^15^ assessment for risk of delirium with a tool called BrainCheck, and receive access to a health coach (HC) through an app called Pip.

The BrainCheck delirium risk screening tool (https://braincheck.com/) is used in the CPC to understand a patient's cognitive status before surgery, to help identify patients who are at higher risk for developing delirium postoperatively. This allows us to preempt the condition in some cases, and to ensure appropriate team members are consulted. Pip Care (PipCare; https://www.pipcare.com/) is an interactive digital health platform (DHP) available on iOS (Apple Inc) and Android (Google Inc) operating systems that provides a human HC and assigns patients with daily tasks. Patients were digitally assigned the appropriate clinical pathways by the human HC following the initial HC‐patient intake and the HC's review of the patient's comorbidities from the electronic medical record. A recent publication in Journal of Medical Internet Research found that there was good patient engagement with Pip Care across many ages (mean age 63 [range 20‐84]), with over two‐thirds of patients completing the program. Additionally, Pip Care engagement was associated with reduced hospital LOS.^16^ Pathways in Pip Care included, but are not limited to, diabetes, nutrition management, protein intake, preoperative fitness, hypertension, anemia management, preoperative and postoperative instructions, as well as preparing the home for after surgery.

Some of the new or preexisting health issues that are commonly addressed include anemia (prescribe and manage iron infusions or B12/folate supplementation), weight loss (HC), hypertension (prescribe antihypertensive agents), chronic heart failure (directly connect with cardiologist), obstructive sleep apnea (refer for sleep testing and treatment), smoking cessation (nicotine replacement), alcohol cessation (testing and treatment), mental health disorders (directly connect with therapist), diabetes (directly connect with endocrinologist), and malnutrition (HC). Many patients require assistance with supportive care such as finances, transportation, and postoperative planning. Typically, the CPC aims to see patients at least 2 weeks before surgery to give adequate time for this care coordination.

### Data Source

In this retrospective cohort study, we included adult patients who underwent free‐flap reconstruction for head and neck squamous cell carcinoma (SCC) between 2013 and 2021 at a single tertiary care academic institution. Patients were excluded if the procedural plan was altered to include split thickness skin graft or regional flap alone. This study was approved by the University of Pittsburgh Institutional Review Board.

### Outcomes

Our outcomes of interest included major and minor complications (see Supporting Information S1: Table 1, available online), length of stay in intensive care unit (ICU), LOS, discharge to acute or subacute care, hospital‐free days (HFDs) within the first 90 days of surgery, and overall survival (OS).

### Study Design

Our goal was to compare the outcomes of interest between perioperative care and our institution's standard of care with respect to medical comorbidities. Patients in the institutional standard of care group received all routine preoperative evaluations in the ENT clinic as well as surgical planning through a multidisciplinary tumor board. The perioperative care group included the aforementioned care with the addition of a CPC clinic visit. Medical comorbidities were measured using the Charlson comorbidity index (CCI), a validated measure of 10‐year survival in patients with multiple comorbidities (detailed criteria found in Supporting Information S2: Table 2, available online).^17^ This index was further shown to be a good prognostic indicator for in‐hospital and 1‐year survival.^18^, ^19^ Patients typically receive two points for solid localized tumors. Considering that all patients included in this study met this criterion, we excluded this as part of our CCI score. Patients were stratified by CCI into subgroups derived from the original validation study: CCI = 0, CCI = 1 or 2, CCI = 3, and CCI ≥ 4.^17^ The benefit of perioperative care versus institutional standard of care was assessed within each subgroup, across each outcome of interest. The results display the relative benefit of perioperative care compared to institutional standard of care within each subgroup, as there were no statistical comparisons made between subgroups. We hypothesized that patients with more severe health comorbidity burden would receive the greatest benefit from perioperative care.

### Demographics and Clinical Characteristics

Demographic information includes age, gender (male, female), and race (white, black, and other). Clinical information includes smoking history (binary), alcohol use history (no, yes, and unknown), previous treatment (none, surgery only, chemoradiation [CRT] only, and surgery + CRT), tumor grade (well, moderate, poor, and unknown differentiation), pathological T stage (pT1, pT2, pT3, pT4, and Tx), pathological N stage (pN0, pN1, pN2, pN3, and Nx), surgical margins (negative, positive, and unknown), perineural invasion (no, yes, and unknown), and extracapsular spread (no, yes, and unknown).

### Statistical Analysis

All statistical analyses were performed using STATA SE 17.0 for Mac OS. Descriptive statistics, including proportions, means, and standard deviations (SDs), were used to report demographic and clinical features of the population. Comparison of categorical population characteristics between treatment groups was made using two‐sided Fisher's exact test, and comparison of continuous population characteristics between treatment groups was made using *t* test. Univariate linear, logistic, and Cox proportional hazards regressions were used to test for association between outcomes and demographic and clinical characteristics. Treatment group, CCI, and the interaction between treatment group and CCI were included in all multivariable models, in addition to covariates that were significantly associated with each outcome on univariate analysis. The interaction term (treatment × CCI) was used for post hoc subgroup testing to evaluate the benefit of perioperative care within each CCI subgroup. Power analyses were conducted to determine the sample size required given the current effect size, 80% power, and significance criterion of *α* = .05. The squared multiple‐correlation coefficient (*R* ^2^) was included to account for multiple covariates (see Supporting Information S3: Table 3, available online).

## Results

The final analysis included 148 patients who received free‐flap reconstruction for head and neck SCC (mean age 62.1 years [SD 10.8]; 68% male [n = 100]; 93% white [n = 138]). The majority of patients received perioperative care (56%; n = 83) and the rest received standard of care (44%; n = 65). Patients in the perioperative care group tended to have higher CCI scores, with only one patient in this group having a CCI = 0 versus eight patients with CCI = 0 in the institutional standard of care group (*P* = .05). Patients in the perioperative care group also tended to have disease of higher N stage (*P* = .002). The remaining demographics and clinical characteristics were not significantly different between treatment groups. Additional details are summarized in Table 1.

**Table 1:** Demographics and Clinical Characteristics Comparison Between Treatment Groups

	Institutional standard of care	Perioperative care	
Variables	n = 65	n = 83	*P* value^a^
Age, y, mean (SD)	61.2 (11.9)	62.9 (9.7)	.34
Gender, n (%)			.56
Male	44 (67.7)	56 (67.5)	
Female	21 (32.3)	27 (32.5)	
Race, n (%)			.38
White	59 (90.7)	79 (95.2)	
Black	4 (6.2)	3 (3.6)	
Other	2 (3.1)	1 (1.2)	
Smoking history, n (%)			.40
No	10 (15.4)	18 (21.7)	
Yes	55 (84.6)	65 (78.3)	
Alcohol history, n (%)			.77
No	18 (27.7)	27 (32.5)	
Yes	47 (72.3)	55 (66.3)	
Unknown	0 (0.0)	1 (1.2)	
Comorbidities			**.05**
0	8 (12.3)	1 (1.2)	
1‐2	25 (38.5)	37 (44.6)	
3	14 (21.5)	20 (24.1)	
≥4	18 (27.7)	25 (30.1)	
Primary site, n (%)			.18
Oral cavity	52 (80.0)	71 (85.5)	
Oropharynx	2 (3.1)	2 (2.4)	
Hypopharynx	8 (12.3)	3 (3.6)	
Larynx	3 (4.6)	7 (8.4)	
Previous treatment, n (%)			.90
None	55 (84.6)	70 (84.4)	
Surgery only	4 (6.1)	6 (7.2)	
CRT only	2 (3.1)	1 (1.2)	
Surgery + CRT	4 (6.2)	6 (7.2)	
Tumor grade, n (%)			.39
Moderately differentiated	51 (78.5)	56 (67.5)	
Poorly differentiated	8 (12.3)	19 (22.9)	
Well differentiated	4 (6.1)	5 (6.0)	
Unknown	2 (3.1)	3 (3.6)	
Pathological T stage, n (%)			.49
T1	5 (7.7)	5 (6.0)	
T2	17 (26.1)	15 (18.1)	
T3	10 (15.4)	15 (18.1)	
T4	33 (50.8)	45 (54.2)	
Tx	0 (0.0)	3 (3.6)	
Pathological N stage, n (%)			**.002**
N0	32 (49.2)	45 (54.2)	
N1	9 (13.9)	9 (10.8)	
N2	20 (30.8)	12 (14.5)	
N3	1 (1.5)	15 (18.1)	
Nx	3 (4.6)	2 (2.4)	
Surgical margins, n (%)			.37
Negative	55 (84.6)	75 (90.4)	
Positive	5 (7.7)	6 (7.2)	
Unknown	5 (7.7)	2 (2.4)	
Perineural invasion, n (%)			.44
No	30 (46.1)	38 (45.8)	
Yes	35 (53.9)	42 (50.6)	
Unknown	0 (0.0)	3 (3.6)	
Extracapsular spread, n (%)			.30
No	8 (12.3)	17 (20.5)	
Yes	22 (33.9)	21 (25.3)	
Unknown	35 (53.8)	45 (54.2)	

Abbreviations: CRT, chemoradiation; n, sample size; SD, standard deviation; y, years.

^a^ Values with *P* value < .05 labeled in bold.

### Postoperative Complications

**Figure 1:**
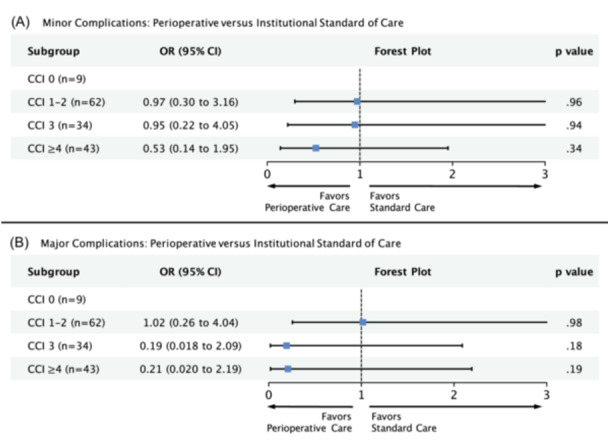
Multivariable model measuring the association between perioperative care and (A) minor or (B) major complications with respect to comorbidity score. Model adjusted for extracapsular extension. 95% CI, 95% confidence interval; CCI, Charlson comorbidity index; OR, odds ratio.

In our multivariable logistic regression model, we adjusted for variables significant on univariate analysis (extracapsular extension, treatment group, CCI, and treatment × CCI). For patients with CCI ≥ 4 (n = 43), we found that odds of minor complication were reduced by almost 50% among those who received perioperative care; however, this result was not statistically significant (odds ratio [OR], 0.53; 95% confidence interval [95% CI], 0.14‐1.95; *P* = .34; Figure 1A). Our power analysis predicts that a sample size of 74 (current n = 43) would be required for the given effect size (Supporting Information S3: Table 3, available online).

Major complications were not significantly associated with any of our measured covariates on univariate analysis. In our multivariable model, we adjusted for treatment group, CCI, and treatment × CCI. We identified nonsignificant reductions in odds of major complications in subgroups CCI 3 (n = 34; OR, 0.19; 95% CI, 0.018‐2.09; *P* = .18) and CCI ≥ 4 (n = 43; OR, 0.21; 95% CI, 0.02‐2.19; *P* = .19; Figure 1B). Our power analysis predicts that for the given effect sizes, a sample size of 250 and 199 would be required for subgroups CCI 3 (current n = 34) and CCI ≥ 4 (current n = 43), respectively (Supporting Information S3: Table 3, available online).

### Length of Stay in ICU

**Figure 2:**
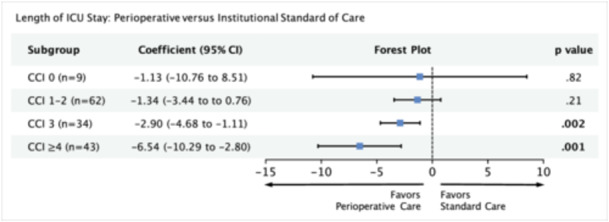
Multivariable model measuring the association between perioperative care and length of intensive care unit stay with respect to comorbidity score. Model adjusted for smoking history and tumor grade. 95% CI, 95% confidence interval; CCI, Charlson comorbidity index; ICU, intensive care unit.

In our multivariable regression model, we adjusted for variables significant on univariate analysis (smoking history, tumor grade, treatment group, CCI, and treatment × CCI). Patients who received perioperative care demonstrated significant reductions in length of ICU stay within the subgroups CCI 3 (coefficient [β], −2.90; 95% CI, −4.68 to −1.11; *P* = .002) and CCI ≥ 4 (β, −6.54; 95% CI, −10.29 to −2.80; *P* = .001). The remaining subgroups demonstrated smaller and nonsignificant reductions in length of ICU stay (Figure 2). For subgroups CCI 1 to 2, our power analysis predicts that a sample size of 270 (current n = 62) would be required for the given effect size (Supporting Information S3: Table 3, available online).

### Length of Hospital Stay

**Figure 3:**
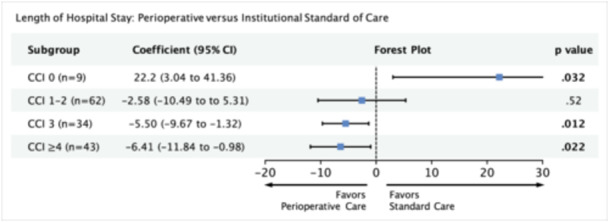
Multivariable model measuring the association between perioperative care and length of hospital stay with respect to comorbidity score. Model adjusted for pathological N stage, surgical margins, and primary site. 95% CI, 95% confidence interval; CCI, Charlson comorbidity index.

In our multivariable regression model, we adjusted for variables significant on univariate analysis (N stage, surgical margins, primary site, treatment group, CCI, and treatment × CCI). Patients who received perioperative care demonstrated significant reductions in LOS within the subgroups CCI 3 (β, −5.50; 95% CI, −9.67 to −1.32; *P* = .012) and CCI ≥ 4 (β, −6.41; 95% CI, −11.84 to −0.98; *P* = .022). In the CCI 0 group, with eight institutional standard of care patients compared to one perioperative care patient, there is a significant increase in LOS for the single perioperative care patient (β, 22.2; 95% CI, 3.04‐41.36; *P* = .032). In the CCI 1 to 2 subgroups, there is a smaller and nonsignificant reduction in LOS (Figure 3). Our power analysis predicts that for the given effect size in subgroups CCI 1 to 2, a sample size of 931 (current n = 62) would be required (Supporting Information S3: Table 3, available online).

### Discharge to Acute or Subacute Care

**Figure 4:**
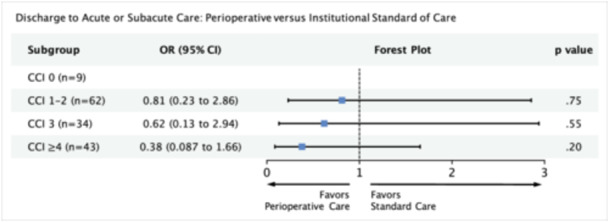
Multivariable model measuring the association between perioperative care and discharge to acute/subacute care facility with respect to comorbidity score. Model adjusted for age, pathological N stage, surgical margins, and perineural invasion. 95% CI, 95% confidence interval; CCI, Charlson comorbidity index; OR, odds ratio.

In our multivariable logistic regression model, we adjusted for variables significant on univariate analysis (age, N stage, surgical margins, perineural invasion, treatment group, CCI, and treatment × CCI). For all subgroups, perioperative care was associated with nonsignificant reductions in odds of being discharged to a facility (Figure 4). Our power analysis predicts that for the given effect sizes, a sample size of 132 and 69 would be required for subgroups CCI 3 (current n = 34) and CCI ≥ 4 (current n = 43), respectively (Supporting Information S3: Table 3, available online).

### HFDs in First 90 Days of Surgery

**Figure 5:**
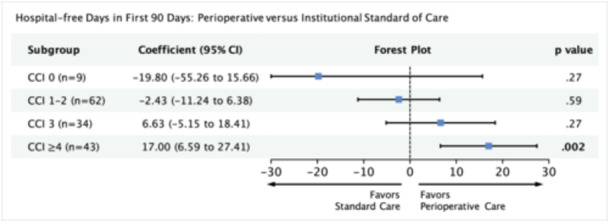
Multivariable model measuring the association between perioperative care and number of hospital‐free days in the first 90 days with respect to comorbidity score. Model adjusted for age and primary site. 95% CI, 95% confidence interval; CCI, Charlson comorbidity index.

In our multivariable regression model, we adjusted for variables significant on univariate analysis (age, primary site, treatment group, CCI, and treatment × CCI). For patients with CCI ≥ 4, we found that perioperative care was significantly associated with a greater number of HFDs (β, 17.00; 95% CI, 6.59‐27.41; *P* = .002). Patients in the CCI 3 subgroup who received perioperative care demonstrated a nonsignificant increase in HFDs (n = 34; β, 6.63; 95% CI, −5.15 to 18.41; *P* = .27). The remaining subgroups demonstrated nonsignificant reductions in HFDs with perioperative care (Figure 5). Our power analysis predicts that for the given effect size in the CCI 3 subgroup, a sample size of 144 (current n = 34) is required (Supporting Information S3: Table 3, available online).

### Overall Survival

**Figure 6:**
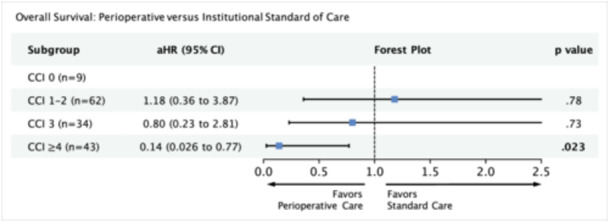
Multivariable model measuring the association between perioperative care and overall survival with respect to comorbidity score. Model adjusted for age, previous treatment, surgical margins, perineural invasion, extracapsular extension, and primary site. 95% CI, 95% confidence interval; aHR, adjusted hazard ratio; CCI, Charlson comorbidity index.

In our multivariable Cox proportional hazards model, we adjusted for variables significant on univariate analysis (age, previous treatment, N stage, surgical margins, perineural invasion, extracapsular spread, primary site, treatment group, CCI, and treatment × CCI). For patients with CCI ≥ 4, we found that OS was improved fivefold among those who received perioperative care compared to those who received institutional standard of care (adjusted hazard ratio [aHR], 0.14; 95% CI, 0.026‐0.77; *P* = .023). Among patients in the CCI 3 subgroup who received perioperative care, there was a nonsignificant improvement in OS (n = 34; aHR, 0.80; 95% CI, 0.23‐2.81; *P* = .73; Figure 6). Our power analysis indicates that for the given effect size in the CCI 3 subgroup, a sample size of 542 (current n = 34) is required (Supporting Information S3 Table 3, available online).

## Discussion

In this study, we evaluated whether perioperative care improves outcomes among patients who received free‐flap reconstruction for head and neck cancer. We found that the benefits gained from perioperative care depend on the severity of preexisting health issues. Patients with greater health comorbidity burden, as determined by CCI, demonstrated significant improvements in length of ICU stay, LOS, HFDs within the first 90 days of surgery, and OS after receiving perioperative care. These results emphasize how perioperative care may be particularly well suited for head and neck reconstruction patients with preexisting health conditions.

Although prehabilitation has been a prominent topic in head and neck literature for the last few decades, the vast majority of studies focus on the associations between preoperative risk factors and surgical outcomes. There is only a small cohort of studies that demonstrate the benefits of some of the preoperative interventions included in CPC. In a recent meta‐analysis published in *Head and Neck*, the authors demonstrated that exercise and nutrition interventions were both independently associated with reduced postoperative hospital LOS among head and neck cancer patients.^20^ In a separate study published in *JAMA Otolaryngology*, the authors found that patients who agreed to be abstinent for a minimum of 7 days preoperatively had significantly lower rates of postoperative alcohol withdrawal, delirium, wound dehiscence, and hospital LOS following head and neck cancer surgery.^21^ Finally, in a large study among surgical patients across several specialties, the authors demonstrated that preoperative glycemic control intervention was associated with lower day of surgery blood glucose and ultimately fewer hospital days following elective surgery.^22^ Overall, these studies highlight that many of the interventions used in the CPC have already been shown to be effective methods of improving outcomes for surgical patients.

In a recent analysis of 1972 head and neck free‐flap patients, Sweeny et al found that higher rates of postoperative complications were significantly associated with several health comorbidities including cardiac disease, pulmonary disease, diabetes, and age > 65.^4^ As management of these health comorbidities is a central component of CPC, we were surprised to find that perioperative care was not significantly associated with rates of major or minor postoperative complications. In our cohort, the most common minor complications were surgical site infection and fistula/wound dehiscence, whereas the most common major complication was return to the OR for hemorrhage or flap failure. A single perioperative care visit may not be sufficient to prevent these types of complications.

Vandersteen et al demonstrated that among head and neck microvascular patients, comorbid health issues are associated with prolonged hospital course.^23^ The observed interaction between CCI and perioperative care in the current study suggests that CCI mediates the benefit of perioperative care for reducing LOS. Other studies have shown that prolonged stays are commonly due to continued nursing requirements and awaiting acute/subacute care placement.^24^, ^25^ Therefore, perioperative care provides an additional opportunity to benefit patients without significant underlying health issues. Perioperative care facilitates preemptive planning of patients' postoperative needs and early coordination of resources, which can potentially prevent discharge delays in all head and neck free‐flap patients.

A recent study by Freeman et al showed that among head and neck free‐flap patients, CCI was associated with a higher rate of acute/subacute care placement.^6^ We did not identify a significant association between perioperative care and rate of discharge to facility. This suggests that perioperative care does not reduce need for acute or subacute rehab, but given the reduced LOS, it may expedite identification of patients that will require additional care at a facility.

HFDs include all days alive that are spent outside of an acute‐care hospital, long‐term acute‐care hospital, or emergency department. Days spent at home, in a long‐ or short‐stay nursing facility, inpatient hospice facility, or rehabilitation facility count as HFDs.^26^ Although data within the head and neck literature are limited, recent ICU data indicate that higher CCI is associated with fewer HFDs.^27^ In the current study, we found that in our CCI of ≥4 or more cohort, patients who received perioperative care spent about 17 fewer days in the hospital within the first 90 days of surgery. The greater number of days spent outside the hospital can be at least partially explained by a shorter LOS. Given the mismatch in effect sizes between these two outcomes, we also suspect that fewer or less lengthy readmissions or emergency room visits have contributed to the increase in HFDs. In a large analysis of head and neck reconstruction patients from the Nationwide Readmissions Database, Goel et al found that 25% of 30‐day readmissions were related to wound complications, 15% were non‐head and neck medical issues, and 25% were due to several other issues including sepsis, pneumonia, bleedings, tracheostomy complication, and electrolyte/nutrition. Further, they identified that several comorbid health issues such as congestive heart failure, COPD, and liver disease were also associated with higher odds of 30‐day readmission.^28^ Although there is profound entanglement between HFDs and several other outcomes addressed in this study, medical comorbidities stand as a unifying risk factor between them all. Overall, perioperative care can directly address comorbid health issues, which are responsible for many of the processes that may cause a patient to return to or remain in the hospital.

For patients who received perioperative care, we found a fivefold improvement in OS in the CCI of ≥4 subgroup. We were not able to evaluate disease‐specific survival, which broadens the differential of mechanisms by which CPC provides a benefit. Given that this benefit is only observed among patients with higher CCI, it is possible that CPC helps connect patients with the necessary resources to longitudinally monitor and manage other potentially lethal health comorbidities. If this finding is mediated through an oncologic mechanism, perhaps CPC helps facilitate receipt of timely surgical and/or adjuvant treatments.

Given the short interval of a few weeks between the CPC visit and surgery, it is surprising that the interventions are associated with acute benefits such as fewer days in ICU and reduced LOS. Although the current study lacks the granularity to bring complete clarity to this question, we suspect that patients may spend additional days in the ICU or hospital for management of issues such as withdrawal, delirium, glucose control, COPD exacerbation, and refeeding. Under normal circumstances, the chronic health conditions that lead to these complications are not cured during an inpatient stay, but rather controlled enough to allow a patient to leave the hospital. Addressing these chronic health conditions preoperatively may allow patients to reach a point of disease control sooner during their hospital stay.

This study has several limitations. Due to the retrospective nature of this analysis, we were unable to capture variables which may have shed more light on the mechanism by which CPC visits are related to fewer days in the hospital or ICU, more HFDs, and improved OS. Of highest priority, in future studies, we hope to identify the specific reasons why certain patients had longer LOS and worse OS without perioperative care. Furthermore, patients were not randomized into treatment groups and therefore there is an unavoidable degree of selection bias. Another limitation that was identified through our power analysis is our small sample size. We were unable to make definitive conclusions regarding the potential benefit of perioperative care across several of our outcomes. Additionally, all outcomes in the CCI 0 subgroup were made between one perioperative care patient and eight patients in the institutional standard of care group. It is unclear whether the single perioperative care patient is generalizable to the target population.

## Conclusions

In this study, we highlighted several benefits of perioperative care among head and neck reconstruction patients. Overall, we found that for patients with higher CCI, perioperative care can reduce length of ICU and hospital stay, increase number of HFDs within the first 90 days of surgery, and improve OS. Although we were unable to identify specific mechanisms for these observations, we speculate that preemptive optimization of comorbid health issues is a major mediator of these postoperative benefits. This conclusion is supported by the observation that the relative benefit of perioperative care tends to improve with increasing CCI. Future studies will be beneficial to delineate which aspect of our perioperative care protocol would be most useful for head and neck patients at different institutions. Implementation of perioperative care may require additional time and resources on the front end of patient care but shows promise in improving patient‐centered outcomes and reducing healthcare costs.

## Author Contributions

**Randall J. Harley**, data curation, formal analysis, methodology, writing—original draft preparation, writing—review and editing; **Stephen A. Esper**, conceptualization, methodology, writing—review and editing; **Tracy Z. Cheng**, conceptualization, methodology, writing—review and editing; **Soukaina Eljamri**, data curation, writing—review and editing; **Preetha Velu**, data curation, writing—review and editing; **Mario G. Solari**, conceptualization, methodology, writing—review and editing; **Shaum S. Sridharan**, conceptualization, methodology, writing—review and editing; **Mark Kubik**, conceptualization, methodology, writing—review and editing; **Seungwon Kim**, conceptualization, methodology, writing—review and editing.

## Disclosures

### Competing interests

The authors declare no conflicts of interest.

### Funding source

The authors have no financial disclosures.

## Supporting information

Supporting Information.

Supporting Information.

Supporting Information.

## References

[1] Dort JC , Farwell DG , Findlay M , et al. Optimal perioperative care in major head and neck cancer surgery with free flap reconstruction. JAMA Otolaryngol Head Neck Surg. 2017;143(3):292‐303. 10.1001/jamaoto.2016.2981 27737447

[2] Lo SL , Yen YH , Lee PJ , Liu CHC , Pu CM . Factors influencing postoperative complications in reconstructive microsurgery for head and neck cancer. J Oral Maxillofac Surg. 2017;75(4):867‐873. 10.1016/j.joms.2016.09.025 27765549

[3] Ishimaru M , Ono S , Suzuki S , Matsui H , Fushimi K , Yasunaga H . Risk factors for free flap failure in 2,846 patients with head and neck cancer: a national database study in Japan. J Oral Maxillofac Surg. 2016;74(6):1265‐1270. 10.1016/j.joms.2016.01.009 26851310

[4] Sweeny L , Curry JM , Crawley MB , et al. Age and comorbidities impact medical complications and mortality following free flap reconstruction. Laryngoscope. 2022;132(4):772‐780. 10.1002/lary.29828 34415067

[5] Parsemain A , Philouze P , Pradat P , Ceruse P , Fuchsmann C . Free flap head and neck reconstruction: feasibility in older patients. J GeriatrOncol. 2019;10(4):577‐583. 10.1016/j.jgo.2018.11.002 30497979

[6] Freeman MH , Shinn JR , Fernando SJ , et al. Impact of preoperative risk factors on inpatient stay and facility discharge after free flap reconstruction. Otolaryngol Head Neck Surg. 2022;166(3):454‐460. 10.1177/01945998211037541 34399644

[7] Lindeborg MM , Sethi RKV , Puram SV , et al. Predicting length of stay in head and neck patients who undergo free flap reconstruction. Laryngoscope Investig Otolaryngol. 2020;5(3):461‐467. 10.1002/lio2.410 PMC7314462 32596488

[8] Vincent A , Sawhney R , Ducic Y . Perioperative care of free flap patients. Semin Plast Surg. 2019;33(1):005‐012. 10.1055/s-0038-1676824 PMC6408252 30863206

[9] Robson A , Sturman J , Williamson P , Conboy P , Penney S , Wood H . Pre‐treatment clinical assessment in head and neck cancer: United Kingdom National Multidisciplinary Guidelines. J Laryngol Otol. 2016;130(S2):S13‐S22. 10.1017/s0022215116000372 27841110 PMC4873895

[10] Healy DW , Cloyd BH , Straker T , et al. Expert consensus statement on the perioperative management of adult patients undergoing head and neck surgery and free tissue reconstruction from the Society for Head and Neck Anesthesia. Anesth Analg. 2021;133(1):274‐283. 10.1213/ane.0000000000005564 34127591

[11] Schmid M , Giger R , Nisa L , Mueller SA , Schubert M , Schubert AD . Association of multiprofessional preoperative assessment and information for patients with head and neck cancer with postoperative outcomes. JAMA Otolaryngol Head Neck Surg. 2022;148(3):259‐267. 10.1001/jamaoto.2021.4048 35050322 PMC8778600

[12] Mahajan A , Esper SA , Cole DJ , Fleisher LA . Anesthesiologists' role in value‐based perioperative care and healthcare transformation. Anesthesiology. 2021;134(4):526‐540. 10.1097/aln.0000000000003717 33630039

[13] Mahajan A , Esper S , Oo TH , et al. Development and validation of a machine learning model to identify patients before surgery at high risk for postoperative adverse events. JAMA Netw Open. 2023;6(7):e2322285. 10.1001/jamanetworkopen.2023.22285 37418262 PMC10329211

[14] Subramaniam K , Boisen ML , Yehushua L , Esper SA , Philips DP , Howard‐Quijano K . Perioperative transthoracic echocardiography practice by cardiac anesthesiologists‐report of a “start‐up” experience. J Cardiothorac Vasc Anesth. 2021;35(1):222‐232. 10.1053/j.jvca.2020.06.046 32888802

[15] Subramaniam K , Subramanian H , Knight J , Mandell D , McHugh SM . An approach to standard perioperative transthoracic echocardiography practice for anesthesiologists‐perioperative transthoracic echocardiography protocols. J Cardiothorac Vasc Anesth. 2022;36(2):367‐386. 10.1053/j.jvca.2021.08.100 34629240

[16] Esper SA , Holder‐Murray J , Meister KA , et al. A novel digital health platform with health coaches to optimize surgical patients: feasibility study at a large academic health system. JMIR Perioper Med. 2024;7:e52125. 10.2196/52125 38573737 PMC11027047

[17] Charlson ME , Pompei P , Ales KL , MacKenzie CR . A new method of classifying prognostic comorbidity in longitudinal studies: development and validation. J Chronic Dis. 1987;40(5):373‐383. 10.1016/0021-9681(87)90171-8 3558716

[18] Quan H , Li B , Couris CM , et al. Updating and validating the Charlson comorbidity index and score for risk adjustment in hospital discharge abstracts using data from 6 countries. Am J Epidemiol. 2011;173(6):676‐682. 10.1093/aje/kwq433 21330339

[19] Radovanovic D , Seifert B , Urban P , et al. Validity of Charlson comorbidity index in patients hospitalised with acute coronary syndrome. Insights from the nationwide AMIS Plus registry 2002‐2012. Heart. 2014;100(4):288‐294. 10.1136/heartjnl-2013-304588 24186563

[20] Seth I , Bulloch G , Qin KR , et al. Pre‐rehabilitation interventions for patients with head and neck cancers: a systematic review and meta‐analysis. Head Neck. 2024;46(1):86‐117. 10.1002/hed.27561 37897197

[21] Kaka AS , Zhao S , Ozer E , et al. Comparison of clinical outcomes following head and neck surgery among patients who contract to abstain from alcohol vs patients who abuse alcohol. JAMA Otolaryngol Head Neck Surg. 2017;143(12):1181‐1186. 10.1001/jamaoto.2017.0553 28447103 PMC5824295

[22] Garg R , Schuman B , Bader A , et al. Effect of preoperative diabetes management on glycemic control and clinical outcomes after elective surgery. Ann Surg. 2018;267(5):858‐862. 10.1097/sla.0000000000002323 28549013

[23] Vandersteen C , Dassonville O , Chamorey E , et al. Impact of patient comorbidities on head and neck microvascular reconstruction. A report on 423 cases. Eur Arch Otrhinolaryngol. 2013;270(5):1741‐1746. 10.1007/s00405-012-2224-z 23081673

[24] Carey MR , Sheth H , Braithwaite RS . A prospective study of reasons for prolonged hospitalizations on a general medicine teaching service. J Gen Intern Med. 2005;20(2):108‐115. 10.1111/j.1525-1497.2005.40269.x 15836542 PMC1490052

[25] Mabire C , Dwyer A , Garnier A , Pellet J . Meta‐analysis of the effectiveness of nursing discharge planning interventions for older inpatients discharged home. J Adv Nurs. 2018;74(4):788‐799. 10.1111/jan.13475 28986920

[26] Auriemma CL , Taylor SP , Harhay MO , Courtright KR , Halpern SD . Hospital‐free days: a pragmatic and patient‐centered outcome for trials among critically and seriously Ill patients. Am J Respir Crit Care Med. 2021;204(8):902‐909. 10.1164/rccm.202104-1063PP 34319848 PMC8534616

[27] Taran S , Coiffard B , Huszti E , et al. Association of days alive and at home at day 90 after intensive care unit admission with long‐term survival and functional status among mechanically ventilated patients. JAMA Netw Open. 2023;6(3):e233265. 10.1001/jamanetworkopen.2023.3265 36929399 PMC10020882

[28] Goel AN , Raghavan G , St John MA , Long JL . Risk factors, causes, and costs of hospital readmission after head and neck cancer surgery reconstruction. JAMA Facial Plast Surg. 2019;21(2):137‐145. 10.1001/jamafacial.2018.1197 30418467 PMC6439803

