# Supplementary material for: Preoperative Care Clinic Improves Survival for Patients Undergoing Free‐Flap Reconstruction
Source: Otolaryngol Head Neck Surg. 2025 Aug 4;173(5):1121–30. doi: 10.1002/ohn.1373 (PMC12574647; doi:10.1002/ohn.1373)
Supplement: Supplementary file 1 — Supporting Information. [file OHN-173-1121-s003.docx]

| **Table S1.** Post-Operative Complications | | |
| --- | --- | --- |
|  |  |  |
| Complications | n | % |
| **Minor** |  |  |
| Wound Infection | 18 | 12.2 |
| Pneumonia | 14 | 9.5 |
| Hematoma | 11 | 7.4 |
| Pulmonary Embolism | 2 | 1.4 |
| Fistula/Dehiscence | 19 | 12.8 |
| Chyle Leak | 0 | 0 |
| **Major** |  |  |
| Return to OR*^a^* | 18 | 12.2 |
| CVA | 0 | 0 |
| ACS | 0 | 0 |
| *Note.* n=sample size; %=percentage; OR=operating room; CVA=cerebrovascular accident; ACS=acute coronary syndrome.  *^a^* Indications include hemorrhage and flap failure. | | |
