# Supplementary material for: Preoperative Care Clinic Improves Survival for Patients Undergoing Free‐Flap Reconstruction
Source: Otolaryngol Head Neck Surg. 2025 Aug 4;173(5):1121–30. doi: 10.1002/ohn.1373 (PMC12574647; doi:10.1002/ohn.1373)
Supplement: Supplementary file 2 — Supporting Information. [file OHN-173-1121-s001.docx]

| **Table S2.** Criteria for Charlson Comorbidity Index | | |
| --- | --- | --- |
|  |  |  |
| Charlson Comorbidity Index |  | Points |
| Age, y | <50 | 0 |
|  | 50-59 | +1 |
|  | 60-69 | +2 |
|  | 70-79 | +3 |
|  | $\geq$80 | +4 |
| Myocardial infarction |  | +1 |
| Congestive heart failure |  | +1 |
| Peripheral vascular disease |  | +1 |
| Cerebrovascular accident |  | +1 |
| Dementia |  | +1 |
| COPD |  | +1 |
| Connective tissue disease |  | +1 |
| Peptic ulcer disease |  | +1 |
| Liver disease | Mild | +1 |
|  | Moderate to severe | +3 |
| Diabetes mellitus | Uncomplicated | +1 |
|  | End-organ damage | +2 |
| Hemiplegia |  | +2 |
| Moderate to severe CKD |  | +2 |
| Leukemia |  | +2 |
| Lymphoma |  | +2 |
| AIDS |  | +6 |
| Solid tumor*^a^* | Localized | +2 |
|  | Metastatic | +6 |
| *Note.* AIDS=acquired immunodeficiency syndrome; CKD=chronic kidney disease; COPD=chronic obstructive pulmonary disease.  *^a^* Not included as criteria in current study. | | |
