# Supplementary material for: Preoperative Care Clinic Improves Survival for Patients Undergoing Free‐Flap Reconstruction
Source: Otolaryngol Head Neck Surg. 2025 Aug 4;173(5):1121–30. doi: 10.1002/ohn.1373 (PMC12574647; doi:10.1002/ohn.1373)
Supplement: Supplementary file 3 — Supporting Information. [file OHN-173-1121-s002.docx]

| **Table S3.** Power Analysis: Sample Size Required for 80% Power | | | | | | |
| --- | --- | --- | --- | --- | --- | --- |
|  | **Minor Complications** | | | | | |
| Subgroup | Current n | P1 (No CPC) | P2 (CPC) | OR | R^2^ | n Needed*^a^* |
| CCI 1-2 | 62 | 0.280 | 0.243 | 0.825 | 0.0441 | 990 |
| CCI 3 | 34 | 0.357 | 0.350 | 0.970 | 0.0441 | 33,108 |
| CCI $\geq$4 | 43 | 0.444 | 0.280 | 0.487 | 0.0441 | 74 |
|  | **Major Complications** | | | | | |
|  | Current n | P1 (No CPC) | P2 (CPC) | OR | R^2^ | n Needed |
| CCI 1-2 | 62 | 0.160 | 0.162 | 1.015 | 0.0491 | 244,682 |
| CCI 3 | 34 | 0.214 | 0.050 | 0.193 | 0.0491 | 250 |
| CCI $\geq$4 | 43 | 0.167 | 0.040 | 0.208 | 0.0491 | 199 |
|  | **Length of ICU Stay** | | | | | |
|  |  | R^2^ (No CPC) | R^2^ (CPC) | ntest | ncontrol | n Needed |
| CCI 0 | 9 | 0.096 | 0.096 | 1 | 2 | - |
| CCI 1-2 | 62 | 0.303 | 0.323 | 1 | 2 | 270 |
| CCI 3 | 34 | 0.231 | 0.449 | 1 | 2 | 23 |
| CCI $\geq$4 | 43 | 0.076 | 0.309 | 1 | 2 | 26 |
|  | **Length of Hospital Stay** | | | | | |
|  |  | R^2^ (No CPC) | R^2^ (CPC) | ntest | ncontrol | n Needed |
| CCI 0 | 9 | 0.083 | 0.744 | 1 | 2 | 8 |
| CCI 1-2 | 62 | 0.284 | 0.290 | 1 | 2 | 931 |
| CCI 3 | 34 | 0.150 | 0.350 | 1 | 2 | 28 |
| CCI $\geq$4 | 43 | 0.260 | 0.370 | 1 | 2 | 47 |
|  | **Discharge to Facility** | | | | | |
|  | Current n | P1 (No CPC) | P2 (CPC) | OR | R^2^ | n Needed |
| CCI 1-2 | 62 | 0.320 | 0.324 | 1.018 | 0.144 | 110,211 |
| CCI 3 | 34 | 0.571 | 0.450 | 0.615 | 0.144 | 132 |
| CCI $\geq$4 | 43 | 0.556 | 0.360 | 0.449 | 0.144 | 69 |
|  | **Hospital-free Days in First 90 Days** | | | | | |
|  |  | R^2^ (No CPC) | R^2^ (CPC) | ntest | ncontrol | n Needed |
| CCI 0 | 9 | 0.163 | 0.599 | 1 | 1 | 10 |
| CCI 1-2 | 62 | 0.0535 | 0.0592 | 1 | 1 | 1298 |
| CCI 3 | 34 | 0.2823 | 0.3201 | 1 | 1 | 144 |
| CCI $\geq$4 | 43 | 0.0278 | 0.1976 | 1 | 1 | 40 |
|  | **Overall Survival** | | | | | |
|  |  | Event Prob | HR | R^2^ | SD | n Needed |
| CCI 1-2 | 62 | 0.210 | 1.02 | 0.257 | 0.491 | 532,169 |
| CCI 3 | 34 | 0.353 | 0.59 | 0.390 | 0.492 | 542 |
| CCI $\geq$4 | 43 | 0.302 | 0.18 | 0.2859 | 0.493 | 51 |
| *Note.* n=sample size; P=probability of event; OR=odds ratio; R^2^=multiple-correlation coefficient; ntest=number of covariates tested; ncontrol=number of adjusting covariates; HR=hazard ratio; SD=standard deviation.  *^a^* Sample size required for 80% power. | | | | | | |
